# Chemosensory sensitivity reflects reproductive status in the ant *Harpegnathos saltator*

**DOI:** 10.1038/s41598-017-03964-7

**Published:** 2017-06-16

**Authors:** Majid Ghaninia, Kevin Haight, Shelley L. Berger, Danny Reinberg, Laurence J. Zwiebel, Anandasankar Ray, Jürgen Liebig

**Affiliations:** 1 School of Life Sciences, Arizona State University, Tempe, AZ 85287 USA; 2 Division of Entomology, Department of Plant Protection, Gorgan University of Agricultural Sciences and Natural Resources, Gorgan, Iran; 3 Departments of Cell and Developmental Biology, Genetics and Biology, University of Pennsylvania, Philadelphia, PA 19104 USA; 4 Howard Hughes Medical Institute and Department of Molecular Pharmacology and Biochemistry, New York University School of Medicine, New York, NY 10016 USA; 5 Department of Biological Sciences, Vanderbilt University, Nashville, TN 37235 USA; 6 Department of Entomology, University of California, Riverside, CA 92521 USA

## Abstract

Insects communicate with pheromones using sensitive antennal sensilla. Although trace amounts of pheromones can be detected by many insects, context-dependent increased costs of high sensitivity might lead to plasticity in sensillum responsiveness. We have functionally characterized basiconic sensilla of the ant *Harpegnathos saltator* for responses to general odors in comparison to cuticular hydrocarbons which can act as fertility signals emitted by the principal reproductive(s) of a colony to inhibit reproduction by worker colony members. When released from inhibition workers may become reproductive gamergates. We observed plasticity in olfactory sensitivity after transition to reproductive status with significant reductions in electrophysiological responses to several long-chained cuticular hydrocarbons. Although gamergates lived on average five times longer than non-reproductive workers, the shift to reproductive status rather than age differences matched the pattern of changes in olfactory sensitivity. Decreasing sensillum responsiveness to cuticular hydrocarbons could potentially reduce mutually inhibitory or self-inhibitory effects on gamergate reproduction.

## Introduction

Chemosensory-based signaling represents one of the major modalities of animal communication^1^. It includes the use of pheromones to attract mates, mark territories, or regulate the organization of insect societies^2^. Although the olfactory system is often adapted to perceive and/or respond to trace-levels of pheromones (e.g. refs 3 and 4), the sensitivity to these semiochemicals can vary depending on context^5, 6^. If high sensitivity is disadvantageous, the olfactory system should show adaptations to counter such negative effects. Such adaptations may occur in eusocial insects where some individuals inhibit reproduction of colony members through pheromones^7, 8^.

Colonies of ants are defined by their reproductive division of labor similar to other eusocial insects: the brood of reproductive specialists, usually morphological queens, is raised by related, but non-reproductive, female helpers. These helpers, who are morphological workers, are still capable of producing their own offspring in most species. One of the major mechanisms involved in the regulation of worker reproduction is pheromonal inhibition by the queen of the colony^9–12^. However, when inhibitory pheromones are detected, they may not only affect the workers but also the queens through self-inhibition or mutual inhibition in the presence of multiple queens^7, 13, 14^, which, in turn, may lead to chemosensory system adaptations.

One group of odorants that is of major importance to reproductive regulation in social insects is part of the lipid surface of the cuticle, and is collectively known as the cuticular hydrocarbons (CHCs). Such low-to-nonvolatile CHC bouquets are predominantly blends of n-alkanes, methyl-branched alkanes, and alkenes^15^. Furthermore, CHC profiles differ between reproductive queens and workers within colonies^10, 16–19^ and between members of different colonies^20^. These differences are used to identify colony membership and to regulate reproduction among colony members which are both required for efficient colony organization in insect societies (e.g. refs 9, 10, 19–23). Despite the importance of CHCs for colony organization, only a paucity of studies has attempted to unravel the mechanisms underlying the peripheral olfactory detection and downstream responses of CHCs using electroantennography^9, 24^, antennal lobe calcium imaging^25^, single sensillum recording^26^ and tip recording^27, 28^.

We investigated the responsiveness of antennal sensilla to general odorants and long-chained hydrocarbons in Jerdon’s jumping ant, *Harpegnathos saltator*. In this species, worker ovaries remain inactive in the presence of a morphologically distinct, reproductive queen. However, in queenless colonies, some previously non-reproductive workers activate their ovaries to gradually become reproductive workers, termed gamergates, over a period of two to three months^29, 30^. During this transition to fertility, the CHC profile of formerly non-reproductive workers changes to that of established gamergates^29^. Distinct qualitative and quantitative differences between worker and gamergate CHC profiles are hypothesized to be perceived as chemical signals that regulate reproduction and dominance interactions within a colony^29, 31^ and mutually affect the ovarian activity of gamergates.

We demonstrate that olfactory sensory neuron (OSN) responses of female-specific antennal sensilla basiconica of *H*. *saltator* to discrete CHCs decrease with the transition from non-reproductive worker to gamergate. This decrease in responsiveness is in line with the hypothesis of mutual pheromonal inhibition in insect societies with multiple reproductives or pheromonal self-inhibition in response to long-chained cuticular hydrocarbons as general fertility signals.

## Results

### Cuticular hydrocarbons are detected by sensilla basiconica on the antenna

**Figure 1:**
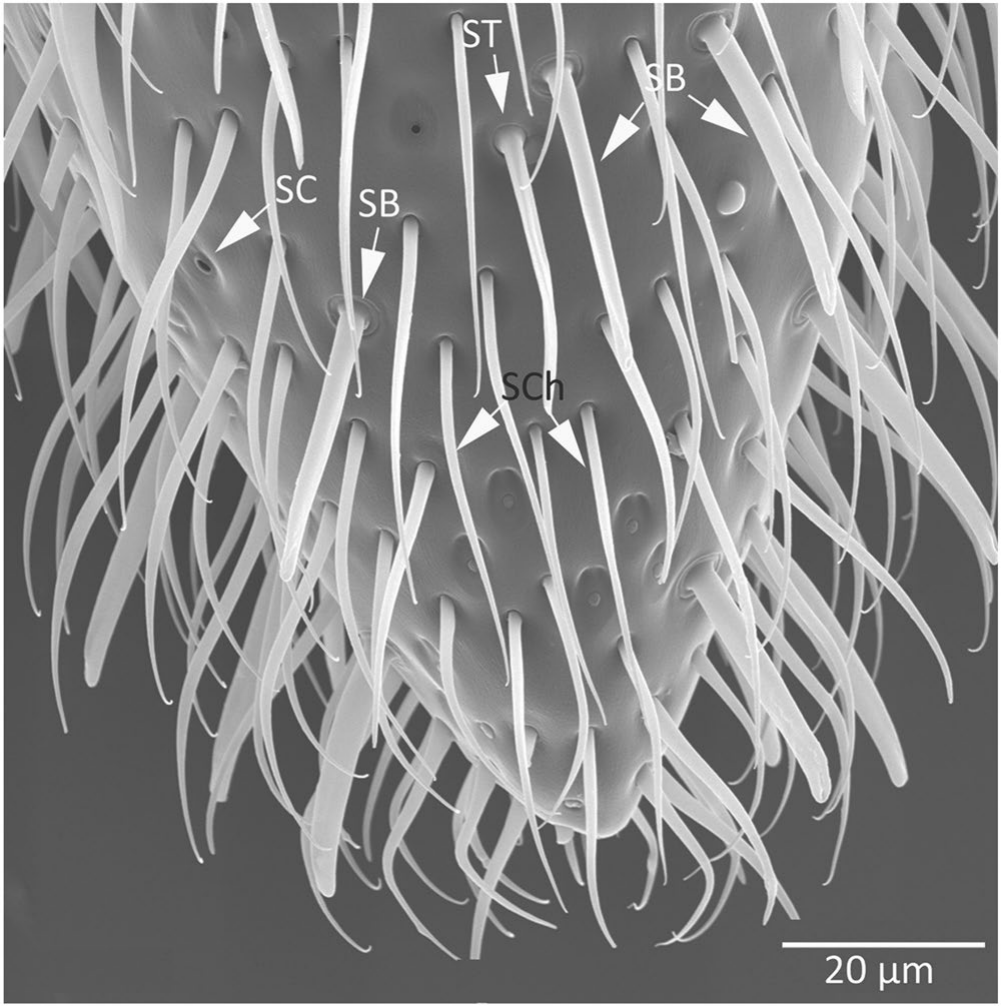
Antennal olfactory sensilla of a female *Harpegnathos saltator*. Scanning electron micrograph showing an overview of a distal segment covered with sensilla basiconica (SB), trichodea (ST), coeloconica (SC), and chaetica (SCh). The latter were not included in the counts as they are not involved in olfaction.

Each antenna of *H*. *saltator* workers and gamergates consisted of 10 flagellomeres which are covered by a number of hair-like cuticular protrusions known as sensilla (Fig. 1). Of the different morphological types of sensilla (coeloconica, trichodea, and basiconica), the sensilla basiconica represent a sex-specific sensillar type present exclusively in females of many ant species^32–34^ including *H*. *saltator*. Overall across a single antenna, approximately 66% of sensilla were s. trichodea, 26% were s. basiconica, and 8% were s. coeloconica with the highest number of s. basiconica present on the last flagellomere in size-matched non-reproductive workers and gamergates (Figs 1 and S1 and Supplementary Tables S1 and S2, Wilcoxon matched pairs test: for each comparison of last flagellomere versus others N = 10, T = 0, p < 0.05 with Bonferroni correction for multiple comparisons). Because it appears that s. basiconica of female ants house a large number of OSNs^32^, the sorting of individual OSN action potentials by amplitude was precluded.

To measure the electrophysiological activity of collective OSNs housed in sensilla basiconica that were mainly present in the terminal antennal flagellomere, we employed the single-sensillum recording (SSR) technique regularly used for insects of varying species, including ants^26, 35–37^. We tested their responses to a panel of 9 methyl-branched alkanes, 20 linear alkanes, and 1 alkene (Z-9-tricosene), ten of which were present in the CHC profile of *H*. *saltator* (Supplementary Table S3). Except Z-9-tricosene, the other hydrocarbons are commonly present in the CHC profiles of other ant species assuming that branched alkanes are represented by R isomeres^15, 38^. To compare these CHC-elicited responses with those elicited by non-HCs, we also included 14 general odors in our panel of compounds (Supplementary Table S3). In order to investigate whether different classes of sensilla may be present among this group of sensilla basiconica, we examined the frequency distribution of responsiveness, where multiple peaks in the distribution would suggest different sensillum types. The frequency distribution of sensillum responses to our panel of 44 odorants did not significantly differ from a normal distribution (Kolmogorow-Smirnov test, d < 0.178, p > 0.2) consistent with sampling from a mostly uniform population of sensilla.

**Figure 2:**
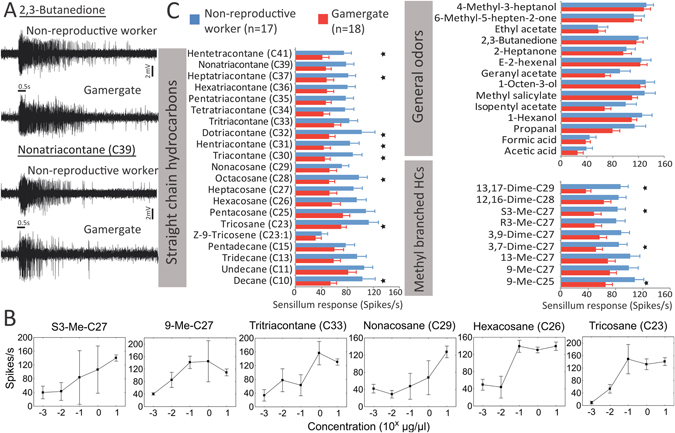
S. basiconica responses of *H*. *saltator* females to an odorant panel. (**A**) Response patterns of antennal sensilla basiconica of a worker and gamergate to 0.5 s non-hydrocarbon (2,3-butanedione) and long-chained hydrocarbon (Nonatriacontane, C39) stimuli. (**B**) Dose response curves for six hydrocarbons for 1 month old workers. N = 4 individuals for concentration 0.001 to 1 µg/µl; N = 5 individuals, n = 18 sensilla for concentration 10 µg/µl based on data from Fig. 4. (**C**) Antennal basiconic SSR responses to 44 hydrocarbons and general odorants in workers and gamergates of *H*. *saltator* with mean and SEM. Stars indicate statistically significant differences between the two groups at p < 0.05 (Repeated measures ANOVA, df 1F > 4.3).

Out of 17 recordings made from non-reproductive workers, all s. basiconica displayed an excitatory signaling mode, albeit with varying response profiles (Fig. 2). With the sole exception of Z-9-Tricosene (C23:1) which evoked only modest responses, all other hydrocarbons tested, including methyl-branched alkanes as well as short- and long-chained alkanes elicited robust responses (Fig. 2). Given our specific interest in CHCs, we established dose-response curves for six compounds for 1 month old workers. The minimum sensitivity is achieved between 10^−3^ and 10^−2^ µg/µl, while a plateau sensitivity is reached between 1 and 10 µg/µl in most cases (Fig. 2B). Therefore, the 10 µg/µl concentration used for all CHC in the non-reproductive worker to gamergate comparison seems adequate. This impression is corroborated by the uniformly strong response to CHCs of our panel. Similarly, apart from the weak to moderate responses to acetic acid, formic acid, and ethyl acetate, strong responses were elicited from the general odors in our test panel (Fig. 2B). Overall, the s. basiconica sampled here displayed a phasic-tonic response dynamic pattern indicated by an abrupt increase in spike frequency that continued well beyond the stimulus delivery and was followed by a decrease in spike frequency (Fig. 2A). Although we observed multiple spike amplitudes corresponding to different OSNs associated with individual sensilla (Fig. 2A), we were unable to distinguish the functional characteristics of the numerous individual OSNs using currently available technology. Therefore, we calculated the summed activity of total OSNs associated with individual sensilla^39^.

### Gamergates show a lower responsiveness to CHCs in sensilla basiconica

In order to examine changes in CHC responses after achieving reproductive status, we next measured the antennal basiconic SSR responses to the hydrocarbons in gamergates without age distinction. The general kinetic characteristics of the responses, including signaling mode, temporal pattern, and the selectivity of the sensilla, resembled those of workers (Fig. 2A). Single sensillum recordings of s. basiconica nevertheless revealed significant differences in the extent of sensilla responses between non-reproductive worker and gamergate s. basiconica (Repeated measures ANOVA, df = 1, F = 4.88, p < 0.05). In fact, the magnitude of sensilla responses to eleven hydrocarbons out of 25 methyl-branched alkanes and straight-chain alkanes with chain length equal or above 23 was significantly lower in gamergates compared to non-reproductive workers (Fig. 2C). In addition, ten out of the remaining 14 hydrocarbons of this group consistently triggered lower levels of responsiveness in s. basiconica that were non-significant, but with a p-value between 0.05 and 0.1 (Repeated measures ANOVA, df = 1, 3.1 < F < 3.9). In contrast, antennal basiconic SSR responses to non-hydrocarbons (general odors) did not differ significantly between gamergates and non-reproductive workers nor were the differences in the p-range of 0.05 to 0.1; in these assays, both non-reproductive workers and gamergates displayed weak-to-moderate responses to acetic acid, formic acid, and ethyl acetate, as well as robust responses to the remaining general odors (Fig. 2C).

Given the consistently lower sensilla basiconica responsiveness to long-chained branched or unbranched alkanes in gamergates, we tested whether the difference between gamergate and non-reproductive worker responsiveness was due to this group of compounds. When testing these hydrocarbons separately, differences between gamergates and non-reproductive workers were still significant (Repeated measures ANOVA: df = 1, F = 6.58, p < 0.05), while the levels of responsiveness to the rest of the odorants together did not show a significant difference between the two groups of workers (Repeated measures ANOVA, df = 1, F = 1.39, p > 0.24).

### Fertility status, not age, underlies reduced responsiveness to CHCs

**Figure 3:**
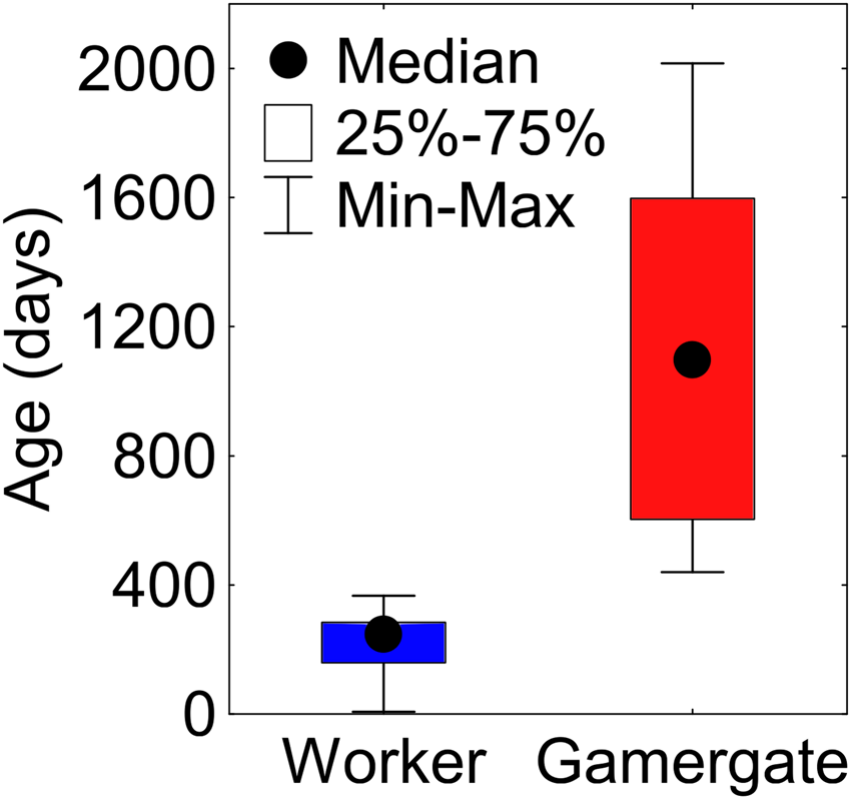
Longevity of *H*. *saltator* females and reproductive status. Differential longevity of gamergates (N = 55) and non-reproductive workers (N = 61) of *H*. *saltator* (t-test, t = 13.4, df = 114, p < 0.00001, data of 21 non-reproductive worker samples were also used in Haight, 2012^43^.

**Figure 4:**
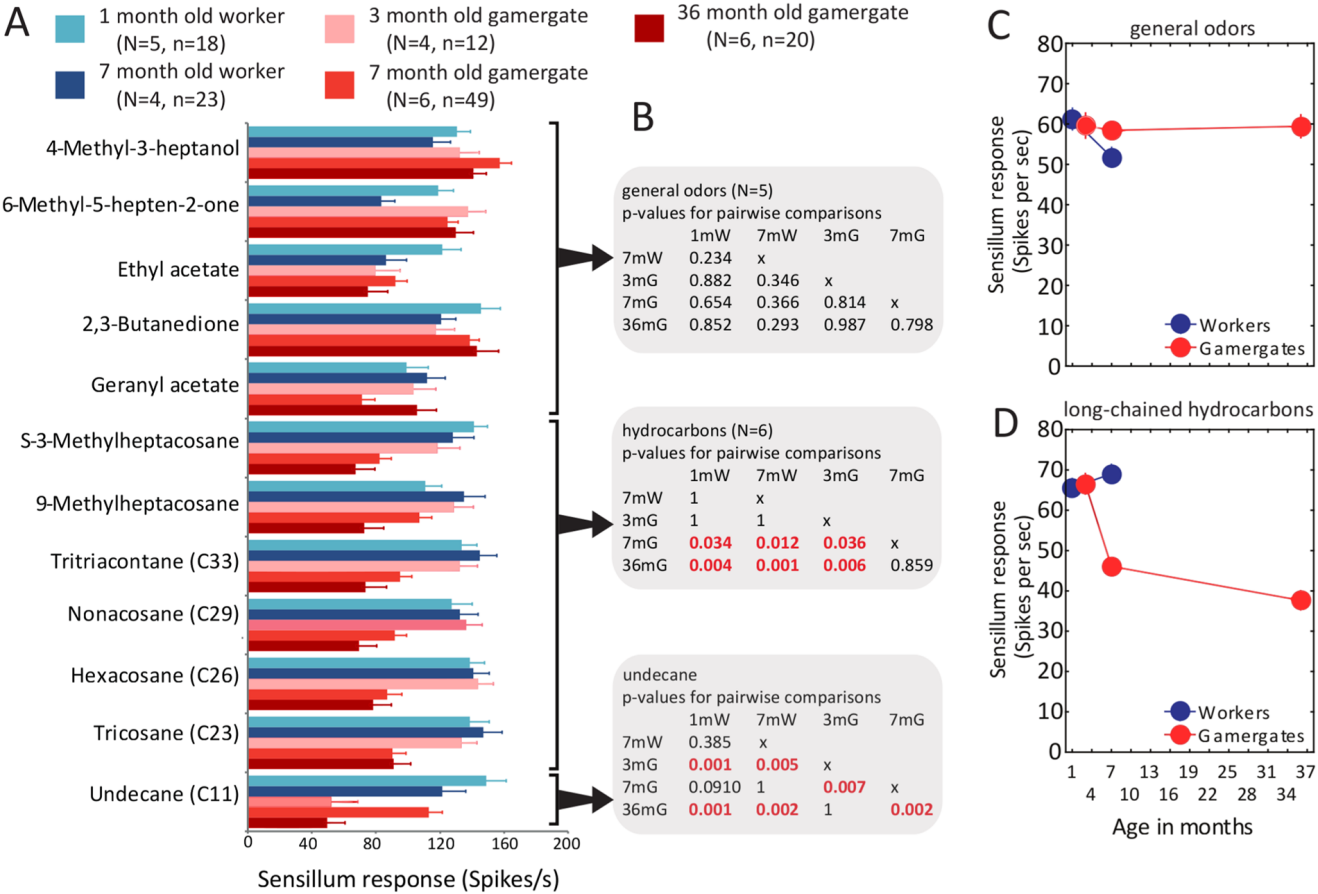
Worker group specific sensillum responsiveness. (**A**) Sensillum responses of non-reproductive workers and gamergates of defined age to a panel of twelve odorants with mean and SEM. (N: number of individuals, n: number of sensilla). (**B**) Differences in the levels of s. basiconica responsiveness to different groups of compounds. Significant differences in the posthoc comparisons are highlighted (Linear mixed model with Fisher’s LSD and Holm-Bonferroni correction) (1 and 7 month non-reproductive workers: 1 mW and 7 mW; 3, 7, and 36 month gamergate: 3 mG, 7 mG, and 36 mG), (**C**) Pooled sensillum responses (mean ± SEM) for compound and fertility and age class from panel c for general odors and (**D**) for long-chained hydrocarbons. Note that error bars do mostly not exceed the size of the marker for the average.

Because gamergates live about five times longer on average than workers (1103 days, N = 55, versus 219 days, N = 61, Fig. 3), the differential response may be related to longevity as well as altered reproductive status. To begin to examine this question, we repeated the sensillum recording with age-controlled workers and gamergates with a smaller panel of 12 compounds representing five general odorants, six long-chained hydrocarbons in the range of compounds typically present on ant cuticles, and undecane, a volatile hydrocarbon (Fig. 4A).

Consistent with what was observed with the larger odorant panel, the s. basiconica responses were normally distributed, and multiple peaks in the distribution were absent (Kolmogorow-Smirnov test, d < 0.086, p > 0.2). The overall comparison among the fertility and age classes showed significant differences in the levels of sensilla basiconica responsiveness (Linear mixed model, df = 4, F = 4.2, p < 0.02). As expected, there was also a significant interaction between group of compounds and fertility and age class (Linear mixed model, df = 8, F = 24.3, p < 0.001). Given the differences in levels of sensilla responses to the three groups of compounds in the previous experiment, we analyzed them separately.

Levels of s. basiconica responsiveness to general odorants did not differ significantly among the non-reproductive workers and gamergates and there was no discernible response pattern (e.g. insofar as age or fertility class) for the volatile short-chained hydrocarbon undecane (Fig. 4A,B). In contrast, the sensillum responses to the group of six long-chained hydrocarbons that were tested were significantly lower in 7- and 36-month old gamergates compared to all other classes of individuals (Fig. 4B). This difference seemed to be due to a reduction of the magnitude of sensillum response at the shift from 3- month to 7-month old gamergates which was after the new reproductive status has been established and accordingly, dominance fights have ceased in the colony (Fig. 4B). When summarizing all sensillum responses for classes of individuals and for compound classes, the average response from sensilla basiconica of both non-reproductive workers and gamergates was very consistent for general odorants (Fig. 4C), but there was an average reduction of 31% in the sensillum responsiveness to long-chained hydrocarbons at the transition from 3-month to 7-month old gamergates (Fig. 4D). Interestingly, this reduction in response in gamergates remained relatively steady for at least 29 months.

### Chronological versus biological age

Given that in *H*. *saltator* colonies, reproductive individuals age differently than non-reproductive individuals like in other social insects (e.g. ref. 40) we also brought biological age differences into this analysis by comparing non-reproductive workers and gamergates at their similar biological age rather than their chronological age (see e.g. ref. 41) The two oldest groups of non-reproductive workers and gamergates were close to their mean longevity (worker: 219 versus 210 days = 7 months; gamergates: 1103 versus 1095 days = 36 months; Fig. 3). In the same light, the youngest age class of non-reproductive workers corresponded to 8.2% of their mean longevity, while the youngest gamergate group attained 13.7% of their mean longevity. Sensillum responsiveness to CHCs in relatively young workers versus relatively young gamergates was not statistically significant, while it was for individuals at their respective mean longevity (Fig. 4D). Responsiveness in gamergates to CHCs did not gradually change with increasing biological age but shifted sharply at an early biological age in gamergates between three and seven months of their life.

## Discussion

We have used physiological tools to analyze the changes occurring in the peripheral olfactory system of *H*. *saltator* during non-reproductive worker to gamergate transitions in order to identify potential adaptations that underlie mutual pheromonal inhibition by reproductive individuals in insect societies. Our data indicate that the peripheral olfactory system of female *H*. *saltator* contains sensory cells that are very sensitive to a range of CHCs. Moreover, the level of responsiveness is plastic, most notably changing with reproductive status. When compared to 7- and 36-month old gamergates, non-reproductive workers regardless of age as well as newly-transitioned 3-month old gamergates displayed antennal OSNs that are on average more responsive to long-chained CHCs. The reduction in responsiveness in older gamergates 4 months after the transition to reproductive status and beyond is consistent with a model in which the inhibitory effects of a CHC-based fertility signals are diminished through adaptations of the olfactory system that reduce pheromonal sensitivity to levels that are consistent with reproductive activity.

Our data demonstrate that the peripheral hydrocarbon detection system in worker and gamergate *H*. *saltator* is plastic which, in turn, could act to reduce the otherwise inhibitory effect of CHC-based fertility signals on ovarian activity in older gamergates. In fact, exposure to a CHC-based fertility signal in a *Lasius* ant or to a volatile queen-derived pheromone in the fire ant *Solenopsis invicta* has been shown to indeed reduce ovarian activity in queens^13, 14^. This type of reproductive regulation has been hypothesized to be a general characteristic in eusocial insects^7, 8^. In *H*. *saltator*, fertile egg production and longevity is an important characteristic of gamergates. Therefore, we expect the evolution of mechanisms that reduce the otherwise inhibitory effect of CHC-based fertility signals on workers that are in the gamergate state. Furthermore, because gamergates remain in the nest and only interact with nestmates, they do not require the same high responsiveness to long-chained hydrocarbons present on insect cuticles as non-reproductive workers. On the other hand, non-reproductive workers are generally involved in colony maintenance, rearing offspring, foraging for food, and monitoring each other’s fertility^31^, tasks where higher responsiveness to CHCs would be beneficial. When workers are newly transitioned from non-reproductive to reproductive status within 2- to 3-months, high responsiveness to CHCs might still be needed (Fig. 2D), as they are still very much involved in frequent dominance interactions and discrimination of small differences in the CHC-based fertility signal of opponents is important^29^. Thus, it is not surprising that responsiveness to our CHC panel was not reduced in 3-month old gamergates who were at the end of the transitionary phase to established gamergate status when their CHC-based fertility signals just became more distinct^29^.

It is important to note, that we cannot exclude the role of compensatory peripheral responses to reduced CHC responsiveness of s. basiconica, or the importance of integrative and other forms of sensory signal enhancement in the ant’s central brain. However, it seems more parsimonious to assume a reduction of general sensitivity to CHCs compatible with the ontogeny of gamergate establishment and the associated benefits of modulating CHC sensitivity rather than invoking a peripheral sensitivity reduction associated with central brain compensation for reduced responsiveness.

## Materials and Methods

### Ants

Colonies of *H*. *saltator* were descendants of colonies collected in 1994, 1995, and 1999 in the western Ghats in southern India^42^ and transferred to the laboratory where they were kept at 25 °C under a 12:12 light:dark cycle. Ants were maintained in closed clear plastic boxes (Pioneer Plastics, Inc., North Dixon, KY, 190 mm long × 135 mm wide 95 mm high) filled with a 25 mm layer of plaster (Modern Materials Labstone Blue, Heraeus Kulzer, LLC., South Bend, IN) with a nest cavity that was covered with a glass plate. Fluon (Northern Products, Inc., Woonsocket, RI) was applied to the insides of the nest box walls to inhibit escapes when the lids were removed. The plaster floor was regularly sprayed with deionized water.

For the determination of status-dependent longevity, non-reproductive workers and gamergates of known ages were sampled from nests established using ants of uniform age. These were created by taking 8–12 ants from their natal colonies on the day they eclosed as adults and placing them together in nest boxes. Twenty such boxes were established using 27 lab colonies as sources. The ants in each box were uniquely wire marked^43^ and provided with small (1^st^ and 2^nd^ instar) larvae in a 1:1 adult: larva ratio. One week after each box was established 1–2 male pupae and 1–2 male adults were provided each box to provide mating opportunities. The boxes were provisioned with crickets twice a week; pre-stung crickets were provided initially until the ants darkened/hardened enough to safely sting crickets for themselves. Individuals in each box were tracked to determine which became gamergates and which remained non-reproductive workers. Individuals were considered to be gamergates if they displayed slow movements within the nest, maintained a zone of space around themselves, exhibited high posture, and were observed laying eggs. Individuals were considered to be non-reproductive workers if they displayed fast movements within the nest, exhibited low posture/deference to known or putative gamergates, and were observed outside the nest. These behavioral observations were made regularly for each individual one month and two months after establishment and haphazardly thereafter.

For the initial comparison of sensillum responsiveness between non-reproductive workers and gamergates, individuals of undetermined age were collected from colonies all originating from the genetic background of colony R22 which was used for genome sequencing^44^. Behavioral determination was the same as for the determination of longevity.

For the age- and reproductive-specific determination of sensillum responsiveness, non-reproductive workers and gamergates were collected from colonies set up as in the experiment for the determination of longevity. One month old workers, however, had eclosed in the same colony where they were wire-marked and later collected for neurophysiological analysis.

### Scanning Electron Microscopy and Analyses of the Antennae

We determined whether numbers of the sensilla basiconica, s. trichodea, and coeloconica differ between the two worker states by analyzing scanning electron microscopy images of whole antenna. Antennae of adult female ants were separated from the head capsule and mounted dorsally, ventrally, or laterally on a specimen holder. The samples were then coated with gold palladium in a Technics Hummer II sputter-coater and imaged in a JEOL JSM-6300 SEM operated at 15 kV. Images of individual flagellomeres were acquired with an IXRF Systems Model 500 digital processor. We counted three distinct sensillum types assumed to be olfactory: trichodea, basiconica, and coeloconica. For the identification of sensilla types, we used the descriptions previously published for other ant species^32^. As only the sensilla on the side facing upward were counted, the counts were doubled to obtain the total number of sensilla per flagellomer segment.

### Preparation of Ants for Electrophysiology

For extracellular recordings, CO_2_-anesthetized ants were placed ventrally on a microscope slide (75 × 25 × 1 mm) that had been covered with a thin layer of modeling clay. To prevent the insects from moving, a piece of clay was put on the dorsal part of the body (except the antennae) and pushed against the underlying clay. The antennae were then gently mounted on the underlying clay and viewed through an Olympus BX51WI light microscope at 60× and 750× magnification, which allowed us to visualize the sensilla of interest for extracellular recordings.

### Extracellular Recordings

Recordings from antennal sensilla basiconica were performed according to the protocols described earlier for other insect species (e.g. refs 36 and 37). For the extracellular recordings we used two electrodes. The ground electrode was a silver wire pierced through the thorax. The recording electrode was a borosilicate capillary glass (World Precision Instruments Inc., FL) that was pulled and sharpened to 1–2 μm tip diameter by an electro puller (Sutter Instrument Co., Model P-2000, CA). The recording electrode was filled with insect ringer’s solution and slipped over a silver wire, and was then gently inserted into the base of a sensillum until electrical contact with sensory neurons was established. Signals were directed to an AC/DC differential amplifier (A-M Systems, Inc., Model 3000, USA) and were subsequently digitized using a digitizer (1440 A Digidata, Axon Devices, USA). Delivery of all the compounds (Supplementary Table S3) was conducted by puffing 2.5 ml of the odor cartridges with a 0.5 s air pulse supplied by a stimulus controller (CS-55, Syntech, The Netherlands) into a constant 89 ml/min humidified main airflow passing over the preparation in a glass tube. Quantification of spikes was carried out offline using Clampfit 10.3 software. We found, while looking through the spike amplitude differences of single recordings, that the sensory neurons housed in single sensilla are too feasibly sorting them out. Therefore, the summed activity of neurons was presented throughout this study. The number of spikes counted during a 0.5 s stimulus delivery period was subtracted from the number of spikes during a 0.5 s prestimulus period, and the outcome was multiplied by 2 to achieve the summed activity of s. basiconicum-associated sensory neurons as a spikes/s measurement for each recording.

### Dose Response Curves

For dose-response curves, six hydrocarbons including S3-Me-C27, 9-Me-C27, Tritriacontane (C33), Nonacosane (C29), Hexacosane (C26), and Tricosane (C23) were presented at increasing doses in linear steps ranging from 0.001 to 10 µg/µl. All the stimuli were tested on individual sensilla. Individual sensilla were always tested from the lowest to highest concentrations. Except for 10 µg/µl, each concentration was tested four times (n_recording_ = 4) on four young individual workers (N_individual_ = 4). The data associated with 10 µg/µl were derived from the large panel. After each run, stimuli were randomized for concentrations 0.001 to 1 µg/µl. Delivery of the compounds and analysis of the responses were done as described above.

### Compounds and Their Delivery Procedure

To assess the physiological activity of OSNs housed in single sensilla, a panel of 30 chemical compounds representing the major classes of CHCs (i.e. n-alkanes and alkenes, and methyl-branched alkanes) as well as 14 general odors were used with either pentane or paraffin oil as solvent at concentrations of 1 or 10 µg/µl applied directly into the glass pipette or onto a piece of filter paper inside the glass pipette, respectively (Supplementary Table S3). We used the same concentrations used in a series of ant papers^26, 45, 46^ so that results could be compared across these studies. Because it is difficult to predict the precise concentration reaching the antenna of an ant in close proximity, antennating and at times touching another ant, or to determine the precise concentration reaching the antenna in our electrophysiology setup, we had added the dose-response curves for some of the most robust ligands identified. Delivery of the compounds was performed according to Ghaninia *et al*.^36^. However, due to their nonvolatile nature at ambient temperature, delivery of CHCs heavier than C23 was facilitated by applying an approximately 0.5 s heat shock using a micro torch after the solvent completely evaporated (BernzOmatic, NY, USA). We excluded any effects of heating on compound integrity by absorbing the delivered compound on an SPME fiber (Supelco, 30 µm polydisiloxane) that was subsequently validated using gas-chromatography together with mass-spectrometry with all parameters as in Moore and Liebig^47^ except that the injection port was set to 260 °C. All compounds produced a single sharp peak that was identical to the non-heated compounds. Although the whole panel of compounds was delivered to the workers and gamergates of unspecified age, and 3- and 36-month old gamergates, only a subset of these compounds was applied to 1- and 7-month old non-reproductive workers, and 7-month-old gamergates. In addition to the delivery of the above-mentioned compounds controls consisting of paraffin oil, water, pentane, and heat were also used. Minor responses to controls were subtracted from those elicited by the odor panel. Responses to controls of the same magnitude as the responses to odorants, however, were criteria to withdraw all recordings from this sensillum.

### Statistical Analysis of Sensillum Responsiveness

The two datasets were analyzed with repeated measures ANOVA and a linear mixed model, respectively. The first dataset consisted of single recordings per individual, while in the second dataset with the reduced compound panel, multiple sensilla per individual were tested. Although both of the datasets were normally distributed, we transformed the dataset for the 44 odorant panel between gamergates and non-reproductive workers using y = ln(80 + x) to reduce heteroscadicity. After transformation, only six compounds deviated significantly from homoscedasticity (Hartley-Cochran-Bartlett test, 0.05 < p < 0.01). Given that the sample sizes of the two groups are nearly equal (Gamergates: N = 18, non-reproductive workers: N = 17), ANOVA is robust to such minor deviations from homoscedasticity^48^. With the transformation, the dataset showed sphericity (Mauchly’s Test of Sphericity: W = 0, Chi-Squ = 973, df = 989, p > 0.63). We applied Duncan’s posthoc test for this dataset. Given that we tested multiple compounds per sensillum, responses to single compounds represent the repeated measurements. The levels of responsiveness to the twelve odorant panel for the age-controlled status comparison (Fig. 2) showed homogeneity of variances (Hartley-Cochran-Bartlett test, p > 0.08). Due to the presence of multiple measurements per individual, we used a linear mixed model with odorant, individual and sensillum nested in individual as random effects, age-specific fertility status and group of odorants as fixed effects, and level of sensillum responsiveness as dependent variable. An identity matrix was chosen for the random effects. Pairwise comparisons were investigated with Fisher’s LSD test and subsequent Holm-Bonferroni correction for multiple comparisons. IBM SPSS 22 (IBM) and STATISTICA 7.1 (StatSoft) were used for the analysis.

## Electronic supplementary material

Supplementary Information
