# Supplementary material for: Chemosensory sensitivity reflects reproductive status in the ant Harpegnathos saltator
Source: Sci Rep. 2017 Jun 16;7:3732. doi: 10.1038/s41598-017-03964-7 (PMC5473913; doi:10.1038/s41598-017-03964-7)
Supplement: Supplementary file 1 — Supplementary Information [file 41598_2017_3964_MOESM1_ESM.pdf]

Chemosensory sensitivity reflects reproductive status in the ant *Harpegnathos saltator*

Majid Ghaninia[1,2], Kevin Haight[1], Shelley L. Berger[3], Danny Reinberg[4], Laurence J. Zwiebel[5], Anandasankar Ray[6], Jürgen Liebig[1,*]

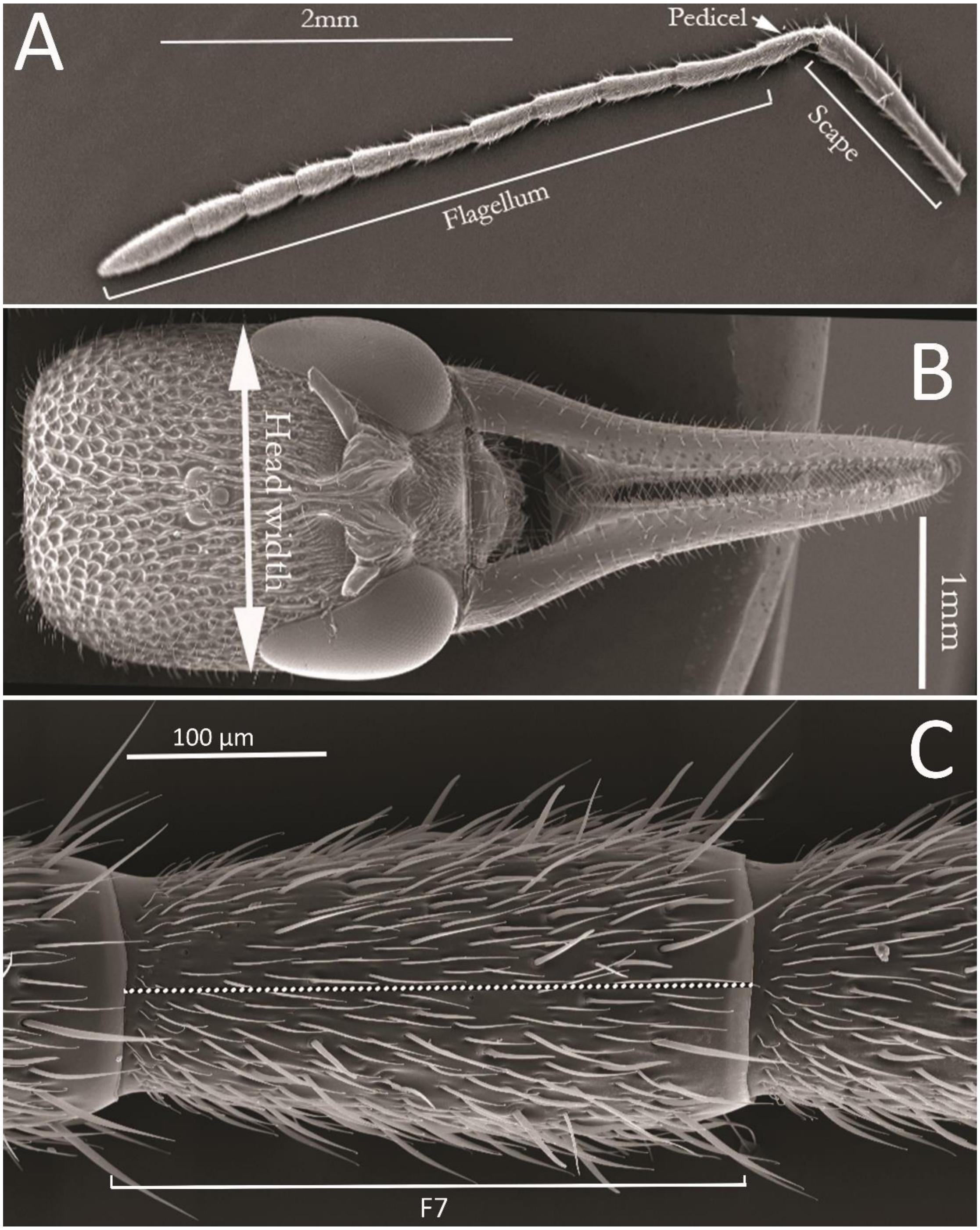

Supplementary Figure S1. Head and antenna characteristics of a worker of *Harpegnathos saltator*. (A) Profile view of the antenna indicating scape, pedicel, and 10 segments of the flagellum. (B) Dorsal view of head (C) Dorsal view of flagellum 7 with dotted line indicating the location of length measurements.

Supplementary Table S1. Number of sensilla in the antennal flagellomeres (F1-F10) of *H. saltator* non-reproductive workers (n=5) and gamergates (n=5) (mean±SE)

Status	Non-reproductive worker			Gamergate		
Sensilla type	Trichoid	Basiconic	Coeloconic	Trichoid	Basiconic	Coeloconic
Flagellomere						
F1	4.8±1.85	2.4±0.75	6.8±1.73	3.2±1.50	2.8±1.02	0.4±0.40
F2	6.8±1.02	3.2±0.49	2.8±0.84	5.2±1.50	3.2±1.02	2±1.10
F3	5.6±1.17	6±1.41	5.2±2.01	5.6±1.72	5.6±1.47	0.4±0.40
F4	8.8±2.24	7.6±0.98	7.2±1.98	10±1.79	8±0.63	1.6±0.40
F5	10.4±2.99	7.6±1.72	4.8±3.17	15.6±2.48	7.6±1.17	1.6±1.17
F6	15.6±0.75	9.6±0.40	6.4±3.16	20.4±2.48	10.8±1.85	2.4±1.47
F7	35.6±4.17	11.6±1.60	6±3.27	38.4±3.06	13.2±1.20	2.8±0.49
F8	51.6±3.49	18±1.41	4±2.23	50±2.76	17.2±2.87	3.2±1.20
F9	74±11.33	18.4±3.66	2±1.39	71.2±8.80	16±1.67	6.8±2.94
F10	143.6±5.27	60.8±8.78	5.6±3.08	143.2±6.05	53.6±4.75	12±3.29
Total	356.8±34.28	145.2±21.20	50.8±22.86	362.8±32.13	138.0±17.65	33.2±12.84

Supplementary Table S2. Size of antennal flagellomeres (F1-F10) of *H. saltator* non-reproductive workers (n=5) and gamergates (n=5) (mean±SE)

Status	Non-reproductive worker	Gamergate
Head width (mm)	2.18±0.04	2.14±0.01
Flagellomere	Flagellomere length (um)	
F1	485.79±6.07	472.25±8.23
F2	440.58±4.37	423.34±6.63
F3	402.01±3.85	394.76±2.50
F4	391.29±3.07	381.58±6.89
F5	368.03±3.53	355.85±3.36
F6	353.96±3.82	341.47±5.33
F7	328.28±4.29	324.15±4.90
F8	313.31±2.33	309.65±3.37
F9	303.83±3.44	299.00±5.17
F10	457.34±6.13	454.35±8.54
Total	3844.45±40.89	3756.44±54.91

Supplementary Table S3. Test compounds and their characteristics for the extracellular recordings of *H. saltator*

Chemical class	Compound name	Heat shock applied	Amount loaded (µl)	Concen-tration (µg/µl)	Source	Cas number	Purity (%)	Solvent
General odor	4-Methyl-3-heptanol		20	10	Sigma-Aldrich	14979-39-6	≥99	Paraffin oil
	6-Methyl-5-hepten-2-one		20	10	SAFC	110-93-0	≥98	Paraffin oil
	Ethyl acetate		20	10	Sigma-Aldrich	141-78-6	99.8	Paraffin oil
	2,3-Butanedione		20	10	Sigma-Aldrich	431-03-8	97	Paraffin oil
	**2-Heptanone***[*]		20	10	Sigma-Aldrich	606-024-00-3	99	Paraffin oil
	***trans*-2-hexen-1-al**		20	10	Sigma-Aldrich	6728-26-3	98	Paraffin oil
	Geranyl acetate		20	10	SAFC	105-87-3	≥98	Paraffin oil
	**1-Octen-3-ol**		20	10	Sigma-Aldrich	3391-86-4	98	Paraffin oil
	**Methyl salicylate**		20	10	Sigma-Aldrich	119-36-8	≥99	Paraffin oil
	**Isopentyl acetate**		20	10	Sigma-Aldrich	123-92-2	≥99	Paraffin oil
	**1-Hexanol**		20	10	Sigma-Aldrich	603-059-00-6	98	Paraffin oil
	**Propanal**		20	10	Sigma-Aldrich	123-38-6	≥97	Paraffin oil
	**Formic acid**		20	10	Sigma-Aldrich	64-18-6	98	Distilled water
	**Acetic acid**		20	10	Fisher	64-19-7	≥99.7	Distilled water
Methyl-branched alkane	**13,17-Dimethylnonacosane (13,17-Dime-C29)**	**	1	1	Synthe-sized[##]	n.a.***	>96.9 [###]	Pentane
	**12,16-Dimethyloctacosane (12,16-Dime-C28)**	**	1	1	Synthe-sized[##]	n.a.***	>89.4 [###]	Pentane
	S-3-Methylheptacosane (S-3-Me-C27)	**	1	1	Synthe-sized[##]	n.a.***	>95.1 [###]	Pentane
	**R-3-Methylheptacosane (R-3-Me-C27)**	**	1	1	Synthe-sized[##]	n.a.***	>95.1 [###]	Pentane
	**3,9-Dimethylheptacosane (3,9-Dime-C27)**	**	1	1	Synthe-sized[##]	n.a.***	>89.6 [###]	Pentane
	**3,7-Dimethylheptacosane (3,7-Dime-C27)**	**	1	1	Synthe-sized[##]	n.a.***	>90.9 [###]	Pentane
	**13-Methylheptacosane (13-Me-C27)**	**	1	1	Synthe-sized[##]	n.a.***	>99.1 [###]	Pentane
	9-Methylheptacosane (9-Me-C27)	**	1	1	Synthe-sized[##]	n.a.***	>97.2 [###]	Pentane
	**9-Methylpentacosane (9-Me-C25)**	**	1	1	Synthe-sized[##]	n.a.***	>99.3 [###]	Pentane

Chemical class	Compound name	Heat shock applied	Amount loaded (µl)	Concen-tration (µg/µl)	Source	Cas number	Purity (%)	Solvent
Straight chain alkane	**Hentetracontane (C41)**	**	1	1	Fluka	7194-87-8	≥99	Pentane
	**Nonatriacontane (C39)**	**	1	1	Fluka	7194-86-7	≥97	Pentane
	**Heptatriacontane (C37)**	**	1	1	Fluka	7194-84-5	≥99.5	Pentane
	**Hexatriacontane (C36)**	**	1	1	Fluka	630-06-8	≥99.5	Pentane
	**Pentatriacontane (C35)**	**	1	1	Fluka	630-07-9	≥99.5	Pentane
	**Tetratriacontane (C34)**	**	1	1	Fluka	14167-59-0	≥99.5	Pentane
	Tritriacontane (C33)[#]	**	1	1	Aldrich	630-05-7	98	Pentane
	**Dotriacontane (C32)**	**	1	1	Aldrich	544-85-4	97	Pentane
	**Hentriacontane (C31)[#]**	**	1	1	Sigma-Aldrich	630-04-6	≥98	Pentane
	**Triacontane (C30)[#]**	**	1	1	Fluka	638-68-6	≥98	Pentane
	**Nonacosane (C29)[#]**	**	1	1	Sigma-Aldrich	630-03-5	99	Pentane
	**Octacosane (C28)[#]**	**	1	1	Fluka	630-02-4	≥99.5	Pentane
	**Heptacosane (C27)[#]**	**	1	1	Sigma-Aldrich	593-49-7	≥98	Pentane
	Hexacosane (C26)[#]	**	1	1	Sigma-Aldrich	630-01-3	99	Pentane
	**Pentacosane (C25)[#]**	**	1	1	Sigma-Aldrich	629-99-2	99	Pentane
	Tricosane (C23)[#]	**	1	1	Aldrich	638-67-5	99	Pentane
	**Pentadecane (C15)**		20	1	Aldrich	*629-62-9*	99+	Pentane
	**Tridecane (C13)**		20	1	Aldrich	629-50-5	≥99	Pentane
	Undecane (C11)		20	1	Sigma-Aldrich	1120-21-4	≥99	Pentane
	**Decane (C10)**		20	1	Sigma-Aldrich	124-18-5	99+	Pentane
Alkene	**Z-9-Tricosene (C23:1)**		20	1	Bedoukian	27519-02-4	98	Paraffin oil
Control	Pentane		20		Sigma-Aldrich	109-66-0	≥99	
	Paraffin oil		20		Sigma-Aldrich	8012-95-1		
	Water		20					
	Blank							
	Heated blank cartridge							

* Underlined bold compounds were not delivered to 1- and 7-month old workers, and 7-month old gamergates
** Heat shock was applied to these compounds
*** Not available
[#] Present on the cuticle of *Harpegnathos saltator* (see Liebig et al. 2000)
[##] For synthesis information see (see Sharma et al. 2015)
[###] Determined by GC/MS, enantiopurity of R- and S-3-methylheptacosane is 99% (see Sharma et al. 2015)
